# N170 Reveals the Categorical Perception Effect of Emotional Valence

**DOI:** 10.3389/fpsyg.2017.02056

**Published:** 2017-11-24

**Authors:** Ruyi Qiu, Hailing Wang, Shimin Fu

**Affiliations:** ^1^Department of Psychology, School of Social Sciences, Tsinghua University, Beijing, China; ^2^School of Psychology, Shandong Normal University, Jinan, China; ^3^Department of Psychology and Center for Brain and Cognitive Sciences, School of Education, Guangzhou University, Guangzhou, China

**Keywords:** N170, categorical perception, emotional valence, faces, event-related potentials (ERPs)

## Abstract

As an important attribute of facial expression, emotional valence has been well explored, but its processing mechanisms remain ambiguous. Investigating the categorical perception (CP) of emotional valence might help uncover the objective basis of the subjective dichotomy of emotional valence and identify the stage at which this processing of valence information might occur. A judgment task was used in the current study with stimuli from the within- or between-category condition, in which participants were required to decide whether two presented faces showed the same emotion. The results of the behavioral experiment revealed a significant CP effect of emotional valence, with faster RTs and greater accuracy for the between- than for the within-category stimuli. In the ERP experiment, the N170 (peaking at approximately 150–170 ms) was found to reflect the CP effect of emotional valence, with a larger amplitude for the within- than for the between-category condition. In contrast, the P1 component (peaking at approximately 100–130 ms) was insensitive to the CP effect of emotional valence. These results reveal the existence of the CP of emotional valence and indicate that the N170 is its earliest electrophysiological index. Therefore, the categorization of emotional valence not only has an objective neural basis but occurs at a relatively early stage of processing.

## Introduction

Facial expression is indispensable in human life, and it plays a crucial role in social interaction. However, the way our brains comprehend this non-verbal language remains a mystery. Studies have shown that recognizing expressions involves different processing stages (Luo et al., 2010; Zhang et al., 2013; Calvo and Nummenmaa, 2016), which suggests that the human brain must accumulate enough information from different aspects (e.g., arousal, threat degree, approachability) of a presented expression before finally understanding it. Emotional valence is one such aspect as it refers to the intrinsic positive or negative character of expressions (Colombetti, 2005) and provides fundamental information for expression recognition (Russell, 2003).

In accordance with the dimensional model (Russell, 2003), which proposed that emotional information of the orthogonal dimensions of affective valence and arousal was perceived initially to assist later processing, behavioral evidence suggests that the processing of valence occurs early and automatically (Lipp et al., 2009; Calvo et al., 2010; McLellan et al., 2010). For example, McLellan et al. (2010) used 100 ms SOA and found priming effects of emotional valence on participants’ response time, this finding is consistent with the opinion that valence information is involuntarily extracted and might serve as an important part of later emotion-related processing (Russell, 2003).

In contrast, ERP studies have produced ambiguous results on the time course of valence processing. Some have found that the P1, N1 and P2 components can be modulated by emotional valence (Smith et al., 2003; Huang and Luo, 2006; Lithari et al., 2010; Zhang et al., 2013; Alguacil et al., 2017). However, other studies have shown that valence can also affect later components, such as LPP (Bernat et al., 2001; Schupp et al., 2004), P3 (Huang and Luo, 2006; Lithari et al., 2010; Alguacil et al., 2017), and LPC (Huang and Luo, 2006). It has been reported that these components are related to differentiation of certain expressions (Luo et al., 2010; Calvo and Beltrán, 2013; Zhang et al., 2013; Calvo and Nummenmaa, 2016), which should occur after valence processing according to the dimensional model. Due to these inconsistent results, it is unclear whether valence information is processed at an early stage.

Categorization is a fundamental property of the human brain. It is a ubiquitous way to simplify input information from the outside world (Harnad, 2003). Categorical perception (CP) also occurs automatically, and we involuntarily lean to categorize stimuli (Maiste et al., 1995; Freeman et al., 2010; Xi et al., 2010). According to Harnad (2003), the CP effect can be operationally defined by between-category separation or within-category compression, i.e., faster RTs or greater accuracy for between- than for within-category stimuli in behavioral experiments. This effect has been found in several stimulus domains, such as color (Fonteneau and Davidoff, 2007; Mo et al., 2011), speech (Liberman et al., 1957; Maiste et al., 1995; Xi et al., 2010), face (Campanella et al., 2000; Morel et al., 2009) and facial expression (Campanella et al., 2002a,b; Kiffel et al., 2005). However, little evidence of the CP of valence information has been reported, though it is a common sense that emotional valence can be categorized. Specifically, it remains to be explored whether the subjective dichotomy of emotional valence has an objective basis and how early this processing can occur.

Because of its excellent time resolution, ERP was used in the present study to explore the time course of the CP effect of emotional valence. The N170 component which peaks approximately 150–170 ms post-stimulus is thought to be sensitive to face and facial expression processing (Schacht et al., 2008; Calvo and Beltrán, 2013; Zhang et al., 2013; Cao et al., 2014). The N170 component has also been found to reflect CP (Campanella et al., 2002a,b; Jacques and Rossion, 2006; Ganis et al., 2012). Campanella et al. (2002b), for example, used morphed emotional faces to test the CP of basic facial expressions and found that the amplitude of the N170 was significantly larger for between- than for within-category stimuli. In addition, previous studies have found that the amplitude of the P1 component is larger and the latency is shorter under between-category conditions (Holmes et al., 2009; Maier et al., 2014). Moreover, evidence indicated that the P1 can also be modulated by complicated stimuli such as faces (Itier and Taylor, 2002, 2004). These results make the P1 an additional ERP component of interest in the current study. Importantly, apart from the objective basis of the emotional valence dichotomy, the present study aimed to explore how early this processing could occur. Previous studies have shown that the CP effect is revealed by both early and relatively late components, which might reflect different levels of categorical information processing (Fonteneau and Davidoff, 2007; Holmes et al., 2009). For example, Holmes et al. (2009) found a CP effect of color on the P1 and the P2, indicating that both early perceptual and later post-perceptual processes are involved in this processing. In the current study, we tested the P1 and the N170 to explore whether the categorization of emotional valence depends on merely low-level perceptual processes or needs more sophisticated information.

Four basic emotions (happiness, anger, disgust and surprise) were chosen in the present study to constitute the within-valence-category condition and the between-valence-category condition. A “same-different” judgment task was adopted, as in previous studies concerning the CP effect (Bornstein and Korda, 1984; Campanella et al., 2000, 2002b; Liu et al., 2010; Hu et al., 2014), to test whether there is CP of emotional valence. After a behavioral version of the task, ERP was recorded while participants were performing the same task. In the ERP experiment, the mean amplitude of the P1 and the N170 were compared across within- and between-category stimuli to assess the time course of the CP of emotional valence, with the contributions of simple perceptual differences being eliminated between them. We hypothesized that the CP of emotional valence not only exists (that is, there should be RTs or accuracy index showing the CP effect), but also occurs at a relatively early stage, such as the P1 and the N170, in accordance with the idea proposed by the dimensional model (Russell, 2003).

## Materials and Methods

### Stimuli and Apparatus

Four basic emotions, happiness, anger, disgust and surprise were chosen to create between-valence-category pairs and within-valence-category pairs. Anger and happiness were selected to be the center of two expression groups. These representative expressions have often been used in previous studies (D’Argembeau et al., 2003; Hugenberg and Sczesny, 2006; Becker et al., 2007; Neel et al., 2012; Craig et al., 2014; Lipp et al., 2015). In the anger-centered group, happiness and anger belonged to different emotional valences; therefore, this pair of expressions constituted the between-category condition. In contrast, the anger and disgust pair constituted the within-category condition. This set was also applied to the happiness-centered group: anger and happiness constituted the between-category condition, whereas happiness and surprise constituted the within-category condition. Previous studies have shown that surprise is usually considered to be positive (Strauss and Moscovitch, 1981; Shah and Lewis, 2003; Kim et al., 2004), so we chose it as another typical positive expression in the happiness-centered group.

**FIGURE 1:**
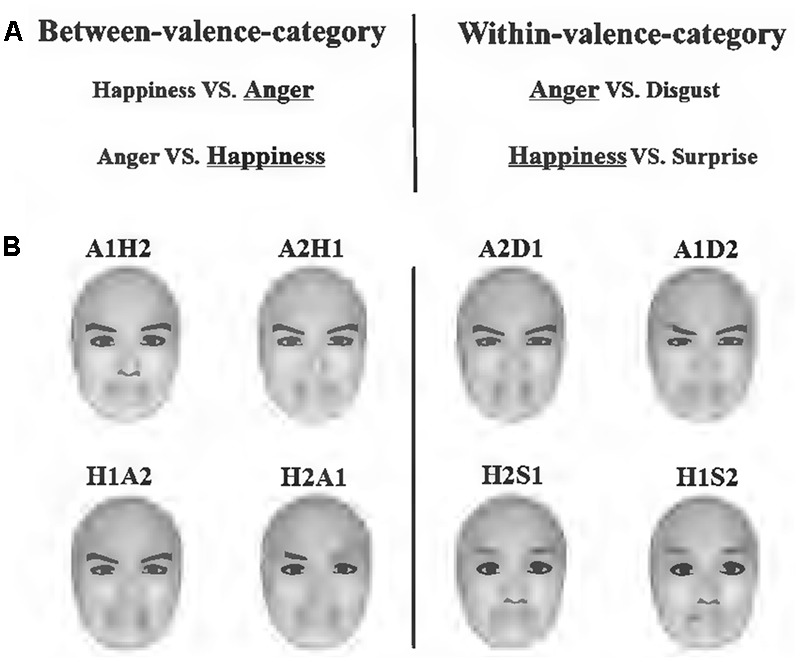
Stimuli and conditions used in the study. **(A)** Emotional expressions chosen to create between-valence-category pairs and within-valence-category pairs. The underlined expressions were centers of different expression groups. **(B)** Illustration of morphed faces used in the experiment. Each face contained two kinds of emotional expressions, e.g., A1H2 was 1/3 anger combined with 2/3 happiness. Faces on the left side belonged to the between-category condition, and those on the right side belonged to the within-category condition.

FaceGen Modeller 3.5 (Toronto, ON, Canada) was used to generate face stimuli. To keep the physical distance between stimuli pairs identical, we blended these emotional expressions. For example, in the anger-centered group, A1D2 was 1/3 anger combined with 2/3 disgust, while A2D1 contained 2/3 anger; A1H2 was 1/3 anger combined with 3/2 happiness, while A2H1 contained 2/3 anger. According to previous studies, the physical distance of the within-valence-category pairs was identical to that of the between-valence-category pairs (Campanella et al., 2002a,b; Harnad, 2003; Kiffel et al., 2005; Roberson et al., 2007). Furthermore, the expressions of happiness generated by the software included smiles both with and without teeth revealed. Happy expressions without teeth revealed were chosen for the anger-centered group, while those with teeth revealed were chosen for the happiness-centered group in order to distinguish the two kinds of stimuli pairs in the between-category condition (both included happiness and anger). All facial images were frontal view and without hair (**Figure 1**).

Stimuli were presented on a 17-inch Viewsonic monitor with a resolution of 1024 × 768 pixels and a refresh rate of 100 HZ. The stimulus presentation was controlled with E-prime 2.0 software (Pittsburgh, PA, United States). All stimuli were presented against a white background at a viewing distance of approximately 60 cm. The stimulus size was 4° × 5.5°(113 × 156 pixels). The center of each stimulus was approximately 3°away from the fixation cross.

### Behavioral Experiment

The main purpose of the behavioral experiment was to examine whether there is CP of emotional valence information and prepare for the following ERP experiment.

#### Participants

A total of 19 healthy undergraduates (13 females), recruited from Tsinghua University, participated in the experiment for fixed monetary reward (50 CNY). Their age ranged between 19 and 22 years, with a mean age of 21 years. All participants were right-handed and had normal or corrected-to-normal vision. The research protocol was approved by the Institutional Review Board, Department of Psychology, Tsinghua University. Written informed consent was obtained from each participant before the experiment.

#### Procedure

The participants were tested in a quiet laboratory room. Two emotional faces were presented simultaneously on the left and right side of a central fixation cross. Each pair of stimuli was preceded by a 500 ms fixation cross and remained until a response was given. There were two blocks of 128 trials. In half of the trials emotional faces were identical on both sides; in the rest, they either both reflected positive/negative expressions or showed expressions belonging to different valence categories. Each face was presented equally often on the left and right sides. Identical pairs were stimuli from within- and between-category conditions and they were used to ensure that participants could press different key buttons. Different pairs of stimuli were randomly intermixed within trial block. Participants were instructed to decide whether those two faces were presenting the same emotional expression. They were told to press the “Z” key if the faces were showing identical expressions, and the “M” key otherwise. Participants were asked to respond as quickly and as accurately as possible. The response button assignments were counterbalanced across participants. A practice block of 27 trials was performed before the experimental session.

#### Results and Discussion

**FIGURE 2:**
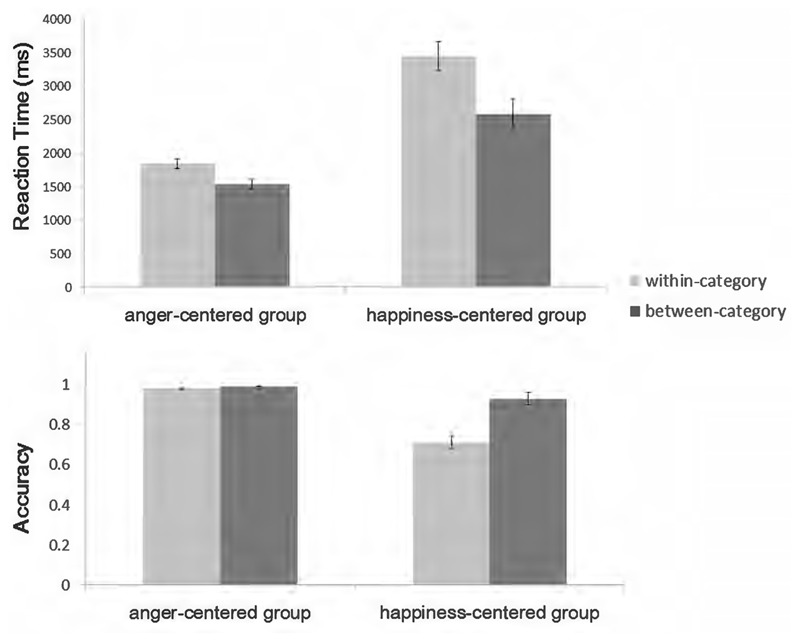
Mean reaction time and accuracy under different conditions.

**Figure 2** summarizes the behavioral results obtained in the experiment. Faster reaction times (RTs) and greater accuracy indicate an advantage for between-categorical differences, which is usually referred as the CP effect. The 2 × 2 repeated-measures ANOVA [valence category (between-category vs. within-category) × emotion group (anger-centered vs. happiness-centered)] on RTs confirmed this impression. Raw RT data were used because the One-Sample Kolmogorov–Smirnov Test showed that they were normally distributed (all *p*-values > 0.05) under each condition and previous studies pertaining to the CP effect have usually analyzed raw data (Campanella et al., 2001; Campanella et al., 2002a,b; Fonteneau and Davidoff, 2007; Holmes et al., 2009; McCullough and Emmorey, 2009; Maier et al., 2014). The results revealed a clear valence category effect (between-category: mean = 2063 ms, *SD* = 928; within-category: mean = 2646 ms, *SD* = 1369), *F*(1,18) = 17.74, *p* = 0.001, $\eta_{p}^{2}$ = 0.496, observed power = 0.978, and a significant main effect for emotion group (anger-centered: mean = 1690 ms, *SD* = 455; happiness-centered: mean = 3019 ms, *SD* = 1340), *F*(1,18) = 33.79, *p* < 0.001, $\eta_{p}^{2}$ = 0.652, observed power = 1.0, with faster RTs for the anger-centered group. There was a significant interaction between valence category and emotion group, *F*(1,18) = 5.004, *p* < 0.05, $\eta_{p}^{2}$ = 0.218, observed power = 0.562. For the anger-centered group, the RT was shorter for the between- than for the within-category pairs (between-category: mean = 1542 ms, *SD* = 364; within-category: mean = 1839 ms, *SD* = 497), *F*(1,18) = 5.51, *p* < 0.05, $\eta_{p}^{2}$ = 0.234, observed power = 0.603. For the happiness-centered group, the RT also reflected an advantage for the between-category pairs (between-category: mean = 2585 ms, *SD* = 1031; within-category: mean = 3454 ms, *SD* = 1493), *F*(1,18) = 13.74, *p* < 0.05, $\eta_{p}^{2}$ = 0.433, observed power = 0.939.

The same analysis was carried out on accuracy. There were significant main effects for valence category (between-category: mean = 96%, *SD* = 0.064; within-category: mean = 84%, *SD* = 0.21), *F*(1,18) = 20.66, *p* < 0.001, $\eta_{p}^{2}$ = 0.534, observed power = 0.990, and emotion group (anger-centered: mean = 98%, *SD* = 0.025; happiness-centered: mean = 82%, *SD* = 0.33), *F*(1,18) = 30.84, *p* < 0.001, $\eta_{p}^{2}$ = 0.631, observed power = 0.999, as well as the valence category × emotion group interaction, *F*(1,18) = 19.34, *p* < 0.001, $\eta_{p}^{2}$ = 0.518, observed power = 0.986. Further analysis showed that for the happiness-centered group, the accuracy of between-category pairs was greater than that of within-category pairs (between-category: mean = 93%, *SD* = 0.077; within-category: mean = 71%, *SD* = 0.23), *F*(1,18) = 20.39, *p* < 0.001, $\eta_{p}^{2}$ = 0.531, observed power = 0.989, while this advantage disappeared for the anger-centered group.

Consistent with the operational definition of CP (Harnad, 2003), we observed CP of emotional valence information, with faster RTs and greater accuracy for the between- than for the within-category condition. However, as the significant interaction effect on RTs and accuracy indicated, discriminability was harder for the happiness-centered group, especially under the within-category condition. This might be related to the categorization advantage of positive expressions (Leppänen and Hietanen, 2003, 2004, 2007; Hugenberg and Sczesny, 2006; Becker et al., 2007; Bijlstra et al., 2010; Craig et al., 2014; Lipp et al., 2015). Positive expressions are too easily categorized as positive, which makes the positive category salient and causes subsequent perception and discrimination to be debilitated and biased (Corneille et al., 2004; Hugenberg and Sacco, 2008). This led to slower RTs and lower accuracy in the present study. Considering the extremely high accuracy in both conditions, the insignificant effect on accuracy for the anger-centered group is probably due to ceiling effects.

Since the phenomenon was observed in both the anger-centered group and the happiness-centered group, all stimuli (including identical pairs) used in the behavioral experiment were kept for the following ERP experiment, in which we examined only the factor of valence category.

### ERP Experiment

After we had found the CP of emotional valence information in the behavioral experiment, the next step was to explore the time course of its associated brain processing.

#### Participants

Eighteen right-handed healthy undergraduates (10 females) from Tsinghua University with normal or corrected-to-normal vision took part in the experiment. Their age range between 18 and 22 years, with a mean age of 20 years. The research protocol was approved by the Institutional Review Board, Department of Psychology, Tsinghua University. Written informed consent was obtained from each participant before the experiment, and all participants were paid a fixed amount of 100 CNY for their participation.

#### Procedure

Participants were seated in a dimly lit, sound-attenuated, electrically shielded chamber. They were asked to focus on the central fixation cross during the presentation of five consecutive blocks of 128 trials each. The task and procedures were similar to those in the behavioral experiment, and identical pairs were used to ensure that participants could press different key buttons. Each trial began with a central fixation cross for 500 ms. Then, two emotional faces were presented simultaneously for 500 ms. Following the stimuli was an interval that varied randomly from 500 to 1000 ms. Participants had to respond as quickly and as accurately as possible. A sequence of 24 stimuli pairs served as a practice trial.

#### EEG Recording and Analysis

The electroencephalogram (EEG) was recorded with Ag/AgCl electrodes (NeuroScan, El Paso, TX, United States) at 64 scalp sites according to the international 10–20 system, with a physical reference electrode located between CZ and CPZ. The horizontal electrooculogram (EOG) was recorded from two electrode sites at the outer canthi of each eye, and the vertical EOG was recorded from electrodes situated on the infra-orbital and supra-orbital regions of the left eye. EEG and EOG recordings were collected with a bandpass of 0.05–100 HZ, and the sampling rate was 500 HZ. Electrode impedance was kept below 5 KΩ.

All channels were filtered with a range of 0.1–30 HZ and re-referenced offline to an average of all scalp electrodes. The EEG analyzing window was between -200 and 600 ms, with 200 ms pre-stimulus EEG serving as the baseline. Ocular artifacts were detected by MATLAB arithmetic and epochs with potentials exceeding ± 75 μV were rejected automatically. Data were segmented and averaged according to valence category, i.e., the within-category and the between-category. Three pairs of channels in the occipito-temporal areas (P7/P8, PO5/PO6, PO7/PO8) were selected for statistical analysis, based on previous studies that show maxima amplitude on these electrodes over both hemispheres (Righart and De Gelder, 2008; Schacht et al., 2008; Utama et al., 2009; Fu et al., 2012). A 2 × 2 × 3 repeated-measures ANOVA [valence category (between-category vs. within-category) × hemisphere (left vs. right) × electrode sites (P7/P8 vs. PO5/PO6 vs. PO7/PO8)] on the amplitude of each component was conducted. *P*-values were corrected by Greenhouse–Geisser correction when necessary.

To exclude the possible influence of the physical attributes of face images, we also analyzed ERPs elicited by the same pairs with identical facial expressions. These pairs of stimuli were separated into two groups according to whether they were used in the between-category condition or in the within-category condition. The same ANOVA analysis described above was applied.

#### Results

##### P1 component

**FIGURE 3:**
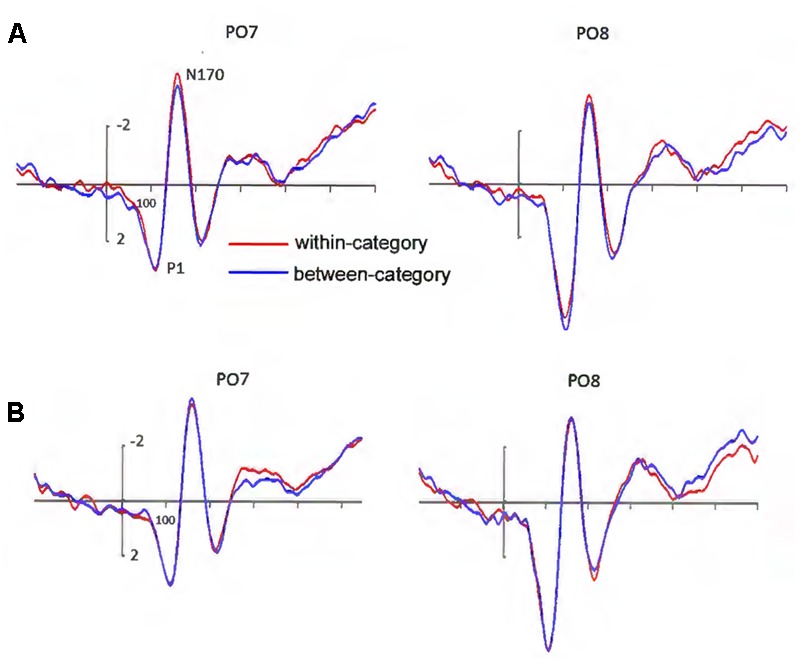
**(A)** Grand-averaged ERPs elicited by within-category and between-category stimuli. The amplitude of the N170 was larger under the within-category condition. **(B)** Grand-averaged ERPs elicited by the same pairs, they were separated according to in which condition these stimuli were used. The amplitude of the N170 elicited by the same pairs did not show valence category effect.

A main effect of hemisphere was observed (left: mean = 2.94 μV, *SD* = 1.86; right: mean = 5.06 μV, *SD* = 2.89), *F*(1,17) = 11.31, *p* < 0.05, $\eta_{p}^{2}$ = 0.399, observed power = 0.886, with the amplitude of P1 being larger over the right than over the left hemisphere (**Figure 3A**). There was also a significant effect of electrode sites (P7/P8: mean = 2.99 μV, SD = 2.45; PO5/PO6: mean = 4.63 μV, *SD* = 2.59; PO7/PO8: mean = 4.37 μV, *SD* = 2.63), *F*(1,17) = 19.50, *p* < 0.001, $\eta_{p}^{2}$ = 0.709, observed power = 1.0, but no main effect for valence category. No other main effects or interactions were significant. Note that the seemingly apparent P1 difference at PO8 was not significant, *t*(17) = 1.69, *p* = 0.109.

##### N170 component

N170 amplitude showed a significant valence category effect (between-category: mean = -3.83 μV, *SD* = 3.70; within-category: mean = -4.25 μV, *SD* = 3.57), *F*(1,17) = 8.92, *p* < 0.05, $\eta_{p}^{2}$ = 0.344, observed power = 0.804, with a larger N170 for within- than for between-category stimuli (**Figure 3A**). There was a significant main effect of electrode sites (P7/P8: mean = -4.62 μV, *SD* = 3.43; PO5/PO6: mean = -3.61 μV, *SD* = 3.59; PO7/PO8: mean = -3.98 μV, *SD* = 3.76), *F*(1,17) = 4.45, *p* < 0.05, $\eta_{p}^{2}$ = 0.357, observed power = 0.678, showing a larger N170 at P7/P8 and PO7/PO8 than at PO5/PO6. We also found a marginally significant interaction between electrode sites and hemisphere, *F*(1,17) = 3.27, *p* = 0.065, $\eta_{p}^{2}$ = 0.290. Further analysis revealed there were significant differences in N170 amplitude between PO5 and PO7, P8 and PO6, P8 and PO8, and PO6 and PO8 (all *p*-values < 0.05). No other main effects or interactions were significant.

As shown in **Figure 3B**, the N170 elicited by the same pairs revealed no significant effect for valence category, indicating that mere differences of physical attributes could not explain our results. There was only a significant main effect for electrode sites (P7/P8: mean = -4.67 μV, *SD* = 3.37; PO5/PO6: mean = -3.52 μV, *SD* = 3.69; PO7/PO8: mean = -3.90 μV, *SD* = 3.87), *F*(1,17) = 5.137, *p* < 0.05, $\eta_{p}^{2}$ = 0.391, observed power = 0.744. No other main effects or interactions were significant.

## General Discussion

The mechanism of emotional valence processing was ambiguous, even though this issue has been widely explored. It remains to be investigated whether the subjective dichotomy of emotional valence has an objective basis and at which stage this processing might occur. In the present study, we focused on the CP effect of emotional valence to address this issue. We observed an obvious CP effect in the behavioral experiment, and the N170 component had a larger amplitude in the within- than in the between-category condition. These results not only confirm the objective basis of the dichotomy of emotional valence, but also provide additional support for the view that the processing of emotional valence occurs at a relatively early stage (Russell, 2003). Furthermore, compared with the insignificant results on the P1, the effect shown on the N170 indicated that fairly more complicated information was needed for categorization of emotional valence whereas merely low-level perceptual processes were insufficient. This finding aligns with previous evidence showing that the N170 reflects some sophisticated processes (Batty and Taylor, 2003; Blau et al., 2007; Krombholz et al., 2007).

Whether the N170 reflects the processing of facial expressions is controversial, and the present study may shed some light on this issue. Traditionally, the N170 was thought to merely index face-specific structural encoding (Eimer, 2000; Itier and Taylor, 2004; Rellecke et al., 2013; Wang et al., 2015; Wang et al., 2016), consistent with the two-stage model of face processing which assumed that the encoding of structural information and emotional information occurred independently (Bruce and Young, 1986). However, studies pertaining to this issue have produced incongruent empirical evidence. Some found that the N170 was not affected by facial expressions (Holmes et al., 2003; Ashley et al., 2004; Holmes et al., 2005; Pourtois et al., 2005), whereas others found that facial expressions could modulate the N170 (Batty and Taylor, 2003; Blau et al., 2007; Krombholz et al., 2007). As mentioned above, facial expressions involve information from different dimensions (Russell, 2003). It is possible that the N170 reveals processing of certain dimensions rather than the integral facial expression. This notion might partially explain those inconsistent results and it has been supported by previous studies. For instance, the multi-stage account of facial expression recognition proposed that the N170 reflects the stage of distinguishing emotional and neutral expressions (Luo et al., 2010; Zhang et al., 2013; Calvo and Nummenmaa, 2016). Furthermore, the N170 was shown to be modulated by valence (Hietanen and Astikainen, 2013; Alguacil et al., 2017). For example, Hietanen and Astikainen (2013) used an affective priming paradigm and found that the amplitude of N170 elicited by neutral faces was affected by the valence of primes. The current study extended past work by providing direct evidence of the N170 reflecting the CP effect of emotional valence, indicating that the N170 component reflects the categorization of emotional valence, which is crucial for further stages of emotional processing of faces (Russell, 2003). Therefore, in addition to structure encoding, the N170 is also involved in facial expression processing, especially for the early processing of emotional valence.

As a well-known face-related ERP component, the N170 was also found to reflect the categorization of different aspects of facial information (Campanella et al., 2000, 2002a,b; Jacques and Rossion, 2006; Ganis et al., 2012), including face identities and facial expressions. Interestingly, however, Research has not yet considered the influence of emotional valence. Campanella et al. (2002b) used morphed faces to explore the CP effect of two basic expressions: happiness and fear. They found that the amplitude of the N170 component elicited by the second facial image decreased in the within-category condition. Importantly, two expressions chosen in that study apparently belong to different valence categories, such that the N170 effect might be contaminated by the CP effects of emotional valence. The present study directly explored the CP effect of emotional valence while keeping the physical distance between pairs identical. The results showed an effect on the N170 component: the amplitude of the N170 component was significantly larger for within- than for between-category stimuli. This effect on the N170 component seems to reverse the pattern of the previous study (Campanella et al., 2002b), but the important differences in the experimental paradigm and the stimuli should be considered between these two studies. First, in the previous study images were presented successively, this manipulation involves a matching procedure and led to a pattern similar to repetition suppression or an adaptation effect (Feng et al., 2013). In contrast, in the current study, the N170 component was more likely to be affected by the difficulty of comparison between different conditions, considering that two images were presented simultaneously. Second, the within-category stimuli from the previous study shared the same expression and the same emotional valence. This set might reduce the mental distance between stimuli more than the present study, in which within-category stimuli shared the same emotional valence but had different expressions. Nevertheless, the present study provides direct evidence that the N170 reflects the CP effect of emotional valence.

No significant CP effect on the P1 was found in the present study, implying that the P1 might be insensitive to the CP of emotional valence. This was different from the results of a previous study, in which the P1 amplitude was found to be significantly higher in the between- than in the within-category condition (Maier et al., 2014). However, this study focused on the influence of the semantic content of verbal categories and used pictures of tools. An oddball paradigm was adopted in their study whereas a matching task was used in the current study, which may to some degree influence the results. Furthermore, the P1 is thought to be mainly sensitive to physical rather than emotional attributes (Calvo and Nummenmaa, 2016). At most it is related to the differentiation between threatening faces and other faces, which must be processed early for self-protection and might involve different processing mechanisms than other expressions do (Luo et al., 2010; Zhang et al., 2013). Therefore, consistent with the finding that the N170 but not the P1 indexes the earliest time for the CP of faces (Ganis et al., 2012), the present results further showed that the N170 but not the P1 indexed the earliest time for the CP of emotional valence.

It should be noted that the results of the present study do not provide direct evidence that the categorization of emotional valence will support later processing of facial expression, and further studies are needed to resolve this issue. Previous studies have found evidence that facial physical properties are enough for expression recognition (Dailey et al., 2002; Susskind et al., 2007) and that valence information might be futile in expression recognition (Calvo and Beltrán, 2013). However, as mentioned above, previous studies have seldom considered emotional valence, and it is inappropriate to eliminate the contribution of this process. Whether and how categorization of emotional valence contributes to expression recognition remains to be explored.

There were several limitations in the present study. Although the CP effect on the N170 was significant, its size was small. This small effect might result from the artificial faces used in the experiment, which were found to influence the amplitude of the N170 (Mühlberger et al., 2009). In addition, the physical distance between the pairs in the current study might be too short. Thus, in further studies real emotional faces should be involved, and the physical distance between pairs should be enlarged to increase this effect. In addition to the small effect, previous studies have found that the N170 component can be modulated by mental resources allocation (Morgan et al., 2008; Senholzi and Ito, 2012; Müller-Bardorff et al., 2016). Thus, amplitude of the N170 under the within-category condition was larger probably because more resources were needed when task difficulty was higher. However, although the difficulty level was also higher for identical pairs than for between-category pairs, there was no significant difference in the amplitude of the N170 across these two conditions. Therefore, variations of task difficulty and mental resources allocation cannot entirely explain the results. However, these confounding factors must be better controlled in future studies.

In sum, the present study substantiated the existence of the CP of emotional valence and indicated that the N170, but not the P1, its neural correlates. The categorization of emotional valence occurs at a relatively early stage of processing.

## Author Contributions

RQ and SF designed the study. RQ collected data. RQ and HW analyzed data. RQ, HW, and SF wrote the paper.

## Conflict of Interest Statement

The authors declare that the research was conducted in the absence of any commercial or financial relationships that could be construed as a potential conflict of interest.
